# Moderating Factors in Culpability Ratings and Rape Proclivity in Stranger and Acquaintance Rape: Validation of Rape Vignettes in a Community Sample

**DOI:** 10.1177/0886260521991294

**Published:** 2021-02-08

**Authors:** Sofia Persson, Katie Dhingra

**Affiliations:** 1 Leeds Beckett University, Leeds, UK

**Keywords:** sexual assault, cultural contexts, date rape, reporting/disclosure, prevention

## Abstract

Rape is a serious concern globally. Past research has identified Ambivalent Sexism (AS), Rape Myth Acceptance (RMA), and the victim–perpetrator relationship as key constructs influencing rape blame attributions and rape proclivity. Limitations with methodologies have, however, limited the practical implications of past research, particularly in the context of underpowered samples and a lack of transparency in vignette development and implementation. In the current research, three studies aimed to validate material to be used in research into rape perceptions and to examine the impact of victim–perpetrator relationship, AS, and RMA on victim and perpetrator culpability, and rape proclivity, using an experimental design. On 563 participants, this research developed and validated six rape vignettes which accounted for methodological limitations of past research (Study One) and were found to be believable and realistic by participants; it further found that aggressively sexist attitudes were associated with increased victim culpability and decreased perpetrator culpability (Study Two), and increased rape proclivity (Study Three). Scenarios of a casual acquaintance produced the highest levels of victim culpability and the lowest levels of perpetrator culpability. Victims were ascribed more control than blame, or responsibility. Men reported the highest levels of rape proclivity in scenarios of casual acquaintance, and intimate partner relationships. Contrary to past research, Benevolent Sexism (BS) did not directly impact attributions in rape cases but may maintain and legitimize the attitudes, which do. As some of our findings contradict past research, we suggest that the need for standardized rape vignettes is evident, along with greater transparency and methodological rigor in sexual assault research, as this will improve the practical implications of findings. Reproducible research practices may be useful for this. While limited in diversity, this research has important implications for policy and research practice, particularly in producing validated material that can be reused by future researchers.

## General Introduction

### Rape

Globally, one in five women will be subjected to sexual assault in their lifetime (García-Moreno, 2005; Rape Crisis UK, 2019). The scope of this problem highlights the importance of examining *how* sexual aggression towards women can be maintained on this scale. Many theories have sought to explain *why* women subjected to rape are uniquely vulnerable for being blamed for their assault relative to victims of other interpersonal crimes (Bieneck & Krahe, 2011; Krahé et al., 2008) and *why* a considerable number of men hold rape-supportive attitudes (Bohner et al., 1998; Eyssel et al., 2006).

Individuals who have been assaulted by someone they know are the least likely to seek support from the police. This is concerning given that 90% of rape perpetrators are known to the victim, either casually or romantically (Black & McCloskey, 2013). Although there are many reasons why victims are reluctant to report acquaintance rape to the police, it has been suggested that a major reporting barrier is the woman’s fear that police will question the veracity and credibility of their claims, or even blame them for the assault. Unfortunately, this concern appears to hold some truth, as a large evidence base has established that a woman who knows her perpetrator is consistently blamed more for the assault than a woman who does not (Gravelin et al., 2018; Suarez & Gadalla, 2010). This is potentially a result of a general societal reluctance to hold men who rape within intimate relationships fully responsible, as illustrated in the delayed criminalization of marital rape (Smith, 2018). However, the evidence is currently inconsistent as to whether there is a linear relationship between level of familiarity and victim blame (Grubb & Turner, 2012; van der Bruggen & Grubb, 2014), perhaps in part due to considerable methodological variations (e.g., different aspects of acquaintance rape vignettes that have been implemented or manipulated) between studies (Grubb & Harrower, 2008). Grubb and Harrower also note that future research needs to expand the acquaintance rape condition (by, for instance, looking at both rape by a partner and an acquaintance), and consider how the above interacts with sexism and rape myths.

### Ambivalent Sexism and Rape Myth Acceptance

Ambivalent Sexism (AS) consists of Benevolent Sexism (BS) and Hostile Sexism (HS), two complementary constructs that form an ambivalently sexist attitude towards women (Glick & Fiske, 1996, 2001). BS encapsulates attitudes that award women a level of dyadic power (e.g., purity, morality, etc.), and appear to work as the kind of “carrot” by which women are awarded for compliance within patriarchy (Chapleau et al., 2007). HS, on the other hand, encompasses negative attitudes towards women and their capabilities, and positions them as seeking to dominate men through deceit and sexual power (Cross et al., 2019; Glick & Fiske, 2001). BS seems to produce higher levels of victim blame in acquaintance rape scenarios, but not in stranger rape scenarios; whereas, HS produces higher victim blame regardless of victim–perpetrator relationship, and correlates with higher rape proclivity among men (Masser et al., 2006; Yamawaki et al., 2007).

Rape myths are persistent and widespread beliefs and attitudes that exonerate the perpetrator and blame the victim of rape (Burt, 1980; Lonsway & Fitzgerald, 1994). Rape Myth Acceptance (RMA) is the degree to which someone believes in rape myths. A large evidence base has found that RMA correlates with victim blame in rape cases, by positioning women as the cause of rape (for a review, refer to Grubb & Turner, 2012), and it seems to play a particularly important role in attributions in acquaintance rape cases (Gravelin et al., 2018; Smith, 2018). It seems to do this through drawing on stereotypes about women as deceitful and childlike, which explains how it links with wider sexism in society (Smith, 2018).

### Methodological Issues in Past Research

There are a number of methodological issues evident in past research in this area, which limit the conclusions that can be drawn from findings.

#### Samples.

Past research has predominantly recruited college-aged student samples (Franklin & Garza, 2018; Newcombe et al., 2008). Our recent meta-analysis (Persson & Dhingra, 2020) examining victim–perpetrator relationships and victim blame noted that 67% of samples were comprised of students, compared to only 14% as comprised of members of the general community. Although no direct comparison has been made on attitudes relating to sexual assault, several researchers have noted that the overall population representativeness of students can be limited when it comes to personality and attitudinal variables (e.g., Hanel & Vione, 2016). It is, therefore, possible that some of the inconsistencies evident in the literature may be partially explained by a lack of clarity about sample limitations and the comparison of student samples with community samples. Although our recent meta-analysis (Persson & Dhingra, 2020) did not find that sample type moderated the effect of victim–perpetrator relationship on victim blame, we note that the relative scarcity of community samples (only five studies recruited community samples), makes this comparison difficult, particularly in the context of significant methodological variation.

In addition, we (Persson & Dhingra, 2020) also noted that few studies reported that power calculations informed sample size determination, and, that most studies were based on a small number of participants and a large number of predictor variables. Consequently, many analyses are likely underpowered. Insufficient power produces serious issues for the accuracy and reproducibility of findings across the social sciences (Ioannidis, 2005), and also limits the practical implications of findings in this area, which is concerning given that research into rape attributions aims to inform and improve provisions for women who have been raped. The current research aims to address the above issues by recruiting well-powered community samples for all three of the studies discussed below. Sample size was determined (and pre-registered) based on *a priori* power calculations using a variety of strategies in the statistical software R (R Core Team, 2020). Moreover, community samples were recruited through Prolific (Prolific.co). In contrast to alternative online recruitment platforms (e.g., Amazon’s mTurk [www.mturk.com]), Prolific pays each participant a minimum wage and is considered to have a wider spread of demographics.

#### Vignettes.

Vignettes are arguably the most common way to research rape attributions (Persson & Dhingra, 2020). Despite their widespread use, there is little data on how believable participants find vignettes in this context, and whether findings can have real-life implications beyond theory. This, therefore, limits the validity of conclusions drawn, and the extent to which findings can be attributed to experimental manipulations (Oppenheimer et al., 2009). This is particularly relevant in the context of acquaintance rape cases; these are perceived as notoriously ambivalent by participants, resulting in an over-reliance on mental heuristics to make attributions (Gerger et al., 2007), which can confound planned manipulations. The lack of validated vignettes is currently a significant gap in past research.

Further, our meta-analysis (Persson & Dhingra, 2020) identified a lack of standardization across the vignettes used to research victim–perpetrator relationships. Specifically, vignettes are often dissimilar in aspects other than the victim–perpetrator relationship, thus impacting on whether findings can be attributed to this variable. For example, many stranger rape conditions (Abrams et al., 2003; Bolt & Caswell, 1981; Calhoun et al., 1976) present the woman as walking home in the evening, without measuring this as an experimental variable. There is evidence to suggest that participants regard this as “risky” behavior, resulting in the attribution of more blame than to a woman who was assaulted by someone known to her. The current research, therefore, develops and validates six sexual assault vignettes using a separate sample, before implementing them experimentally.

#### Measures.

Outcome Measures. Whilst most outcome measures in this area generally tap into perceived victim culpability and precipitation (Gravelin et al., 2018; Hockett et al., 2016), the specific items used tend to vary across blame, responsibility, and control (Persson & Dhingra, 2020). Although evidence is mixed as to whether ratings differ between these different measures (Richardson & Campbell, 1980; Shaver & Drown, 1986), it nonetheless calls for increased measurement consistency, in order to facilitate comparability. Victim and perpetrator culpability measures have, consequently, been specifically developed for the current study, and measure blame, responsibility, and control separately.

Attention Checks. All studies discussed in this article included a variety of pre-registered attention checks. Attention checks (Oppenheimer et al., 2009) are designed to examine whether participants are actually reading the questions, rather than selecting an answer at random, or not reading the instructions. Where participants fail a pre-set threshold of these checks, they are excluded from the final analysis. As noted by Oppenheimer et al. (2009), attention checks can increase the quality of research findings, particularly when manipulating experimental variables. Thus, attention checks—when designed carefully and not used as a replacement for considering confounding variables (Hauser et al., 2018)—can improve the validity of psychological research, and contribute to better reproducibility across the social sciences, which is an urgent concern (Ioannidis, 2005). The current research, therefore, includes carefully designed attention checks throughout.

## The Present Research

In light of the above, the present research aims to provide a comprehensive investigation into the impact of victim–perpetrator relationship on victim and perpetrator culpability, as well as rape proclivity. Through three separate studies, we seek to clarify previous inconsistencies in the overall literature, specifically focusing on the potential for a linear relationship between perceived culpability and relationship-proximity, and whether variations in the stranger rape conditions can negate the effect of the victim knowing the perpetrator. It aims to do this by developing and validating six rape vignettes (Study One), and examining the impact of victim–perpetrator relationship on victim and perpetrator culpability ratings (Study Two), and rape proclivity (Study Three). Combined, this research provides a rigorous examination into moderating factors in victim and perpetrator culpability and rape proclivity; we further signpost recommendations for future research, and provide validated materials that can be used by other researchers.

## Transparency Statement

All materials that were developed specifically in this article (Studies One, Two, and Three) can be found in repositories on the Open Science Framework (https://osf.io/92bq7/).

This includes the pre-registration for this study, and study hypotheses (Studies One, Two, and Three). The full datasets (and code) will be made available through the repository once all analyses on the datasets have been conducted.

## Study One

Study One aims to develop and validate six vignettes depicting a rape of a woman by a man. These vignettes include a number of manipulations that address key limitations in past research, as outlined above.

### Method

#### Design.

This study used an exploratory, repeated-measures design. There were six vignettes in total (Appendix B; https://osf.io/pksqb/), depicting two assaults in each of the following categories: stranger, acquaintance, and partner. Additional variables that were varied were: whether or not the woman engaged in flirtatious behavior prior to the assault (in the partner and acquaintance vignettes) and whether or not she was assaulted in her home, or when walking home at night (in the stranger vignettes). Consequently, participants each viewed three vignettes (one stranger, one acquaintance, and one partner rape), but additional variables were randomized.

##### Data analysis strategy.

Data were analyzed using base R functions in the statistical software environment R (R Core Team, 2020). The sample was split according to which vignettes participants viewed, and frequency tables for all relevant variables were generated. In line with our study pre-registration, 80% was set as the target for acceptability for all of the questions. There was no blinding.

##### Participants.

Sample size was based on an exploratory power calculation conducted in R (R Core Team, 2020). The power analysis (O.H. Clark, personal communication, December 10, 2019) estimated answer distributions in seven different scenarios of hypothetical coin tosses and predicted that a minimum of 50 participants reading each vignette would be required to reach a minimum of 80% manipulation accuracy and credibility for the vignettes. As each participant read three (out of six) vignettes, a minimum of 100 participants were needed. One-hundred and twenty-nine participants were included in the final analysis. Originally, 144 participants completed the study, but 15 were excluded due to failing an attention check. Participants were all UK residents with a mean age of 35.31 years (*median* = 33, *min* =18, *max* = 82). The sample included 70.54% women (*n* = 91), and 29.46% men (*n* = 38). Participants were paid £1.15 for taking part.

##### Materials.

###### Vignette development.

This study validated a total of six rape vignettes. As outlined above, these vignettes were developed to address key limitations of previous vignettes (validity and credibility issues; lack of data on manipulation checks; variable vignette standardizations; potentially unrealistic content) in this area, and also considered recommendations for vignette development in experimental research (Hughes & Huby, 2004; Steiner et al., 2017), drawing on considerations of internal validity, realness, and cognitive demand. Internal validity was achieved by drawing on research expertise and thorough literature reviews in the area (Persson & Dhingra, 2020). Realness was achieved by drawing on the realities of rape, for example, by portraying the assault in line with common features of rape (e.g., without the use of a weapon, the attack taking place in the home, etc.). As names in vignettes previously used in this area (e.g., “Kathy” and “Jason;” Abrams et al., 2003) can be critiqued for sounding out of place within a contemporary UK context, the current vignettes’ victim and perpetrator names were selected to reflect the most common UK names for those in their early 20’s, both nationally and in London (Office for National Statistics [ONS], 2018). The age range for names was chosen to reflect the fact that risk of being subjected to sexual assault is highest for women aged between 18 and 24 years (and perpetrated by one of their peers of similar age), thus reflecting the “typical” victim (ONS, 2019). As other vignette details are relatively context-free, victim and perpetrator names can be altered, so that these vignettes can be implemented in other countries and contexts. Manipulated variables included the victim–perpetrator relationship (three levels: stranger, acquaintance, partner), flirtatious behavior by the victim of a perpetrator she knew (two levels: present, not present), and risky behavior by the victim of stranger rape (two levels: present, not present). Therefore, we initially tested six vignettes: A. Partner—no flirtation; B. Partner—flirtation; C. Acquaintance—no flirtation; D. Acquaintance—flirtation; E. Stranger—home; F. Stranger—risk.

###### Target measures.

Two sets of target measures were developed specifically for this study. Responses were dichotomous (“Yes” or “No”).

###### Manipulation check.

Four items asked participants to identify key details of the vignettes: sex as non-consensual (MC1), the victim–perpetrator relationship (MC2, MC3), and victim flirtatious behavior (MC4). Participants were deliberately asked to identify the incident as non-consensual rather than rape, as past research has indicated that rape is a loaded term (Papendick & Bohner, 2017), and may rely on pre-conceived ideas about rape and general victim-blaming attitudes to inform decision-making (Bohner et al., 2009). Manipulations were not used as participant exclusion criteria, as the purpose of them was to establish the validity of the vignettes, not the validity of the responses.

###### Credibility.

Three items asked participants to indicate how believable (C1) and realistic (C2) they found the vignette, and whether they felt that they could make similar attributions about the vignette as they would do about a similar, real-life case (C3). The final question aimed to account for criticisms of artificiality and limited real-life application which are commonly leveled at vignette research (Donnon et al., 2009).

###### Attention check.

Participants were presented with one attention check item, to ensure the vignettes had been read carefully.

##### Procedure.

Data collection took place on Qualtrics Version XM (2020). After reading the study information, and indicating consent, participants were presented with three vignettes, which were randomized according to the block randomization option in Qualtrics (2020). After each vignette, participants were asked the manipulation check and credibility items, which were randomized in order. Then, participants indicated their gender and their age and were asked the attention check item. The study took about 10 minutes.

##### Ethical considerations.

The study received ethical approval from the local ethics coordinator at Leeds Beckett University and was conducted in accordance with the British Psychological Society’s (BPS) Code of Ethics (2018).

#### Results

#### Manipulation check and credibility.

Detailed results can be found in Table A1 (Appendix C; https://osf.io/92bq7/). Participants identified all key vignette manipulations, apart from one manipulation in one of the partner conditions (vignette B). Even though the vignette included the woman kissing the man prior to the assault, 29.85% of participants did not identify this as flirtatious behavior. Therefore, this manipulation fell short of our acceptance level, and vignettes with this manipulation (flirtatious behavior; vignettes B and D) were dropped from Studies Two and Three. Results were similar for women and men. Vignettes also reached an acceptable level of credibility (>80%), indicating that the accounts were viewed as believable and realistic by participants. Importantly, participants viewed the vignettes as representative enough of rape that they felt able to make similar judgments as they would do about a real-life case.

### Discussion

This study validated six carefully developed rape vignettes. Only one manipulation (victim flirtatious behavior) failed to reach acceptance, and will, therefore, be dropped from Studies Two and Three. It is important to note that our wording of the manipulation check may have been problematic, and asking participants whether the woman had shown any interest in sexual contact, for instance, may have led to a different outcome. This study is the first of its kind to systematically collect data on the quality of key manipulations, as well as to assess credibility of sexual assault vignettes. It, therefore, makes a considerable contribution to the current literature, and has positive implications for future research, particularly in strengthening the methodological rigor of this field.

## Study Two

This study aims to implement four of the vignettes validated in Study One to examine how the victim–perpetrator relationship impacts ratings of victim and perpetrator culpability, and how this is moderated by RMA and AS. Specifically, we examine three victim–perpetrator relationships: partner; acquaintance; and stranger. Accounting for a previous lack of standardization (Persson & Dhingra, 2020) in stranger rape conditions, we test two variations of this condition: a “risky” stranger condition; and a “non-risky” stranger condition, to examine whether this impacts results. Our exploratory analyses also consider whether there are differences within the culpability constructs, that is, whether ratings of blame, responsibility, and control vary across victim and perpetrator in different scenarios.

### Method

#### Design.

This study used a between-subjects design. Four vignettes were used (vignettes A, C, E, and F), as validated in Study One. These consisted of one partner rape vignette, one acquaintance rape vignette, and two stranger rape vignettes (risky *versus* non-risky behavior). Additional predictor variables included BS and HS, RMA, and gender. Outcome variables were victim and perpetrator culpability.

#### Data analysis strategy.

Data were analyzed using various functions in the statistical software environment R (R Core Team, 2020). Three separate regressions were conducted for each of the outcome variables. Impression management was controlled for throughout. The first examined BS and victim–perpetrator relationship as the main predictors (including interaction terms) while controlling for HS. The second examined HS and victim–perpetrator relationship as the main predictors (including interaction terms) while controlling for BS. The second examined RMA and victim–perpetrator relationship as the main predictors (with interaction terms). Main effects were taken from the first model, and any interaction effects were taken from the subsequent models. Effect sizes are reported in Cohen’s *d* (Cohen, 1992), and calculated according to formulas by Cumming (2012).

#### Participants.

Sample size was based on a power calculation conducted in R (R Core Team, 2020), using the “pwr” package (Champely, 2020). The power level was set at 95% with six predictor variables, and the target effect size was small-medium (*f*^2^ = .098), based on our previous meta-analysis in this area (Persson & Dhingra, 2020). The significance value was set at *p* < .05. As illustrated in Figure A1 (https://osf.io/xdavj/), 240 participants were needed.

Two-hundred and fifty-two participants were included in the final analysis. Participants were all UK residents with a mean age of 35.31 years (*SD* = 10.21, *median* = 33, *min* = 18, *max* = 82). The sample included 75% women (*n* = 189), 24.6% men (*n* = 62), and .4% non-binary (*n* = 2) people. The vast majority were heterosexual (85%), and a minority identified as bisexual (10%), gay (4%), or another sexuality not listed (1%). In terms of ethnicity, participants were White (92%), Asian (3.2%), Black British (2.4%), and of Other or Mixed Ethnicity (2.6%). Participants were paid £1.79 for taking part.

#### Materials.

##### Vignettes.

Participants were presented with one of four vignettes: a partner rape (A); an acquaintance rape (C); a stranger rape in the woman’s home (E); and a stranger rape with the woman walking home in the evening (F).

#### Predictor variables.

##### Ambivalent sexism.

AS was measured using the Ambivalent Sexism Inventory (ASI; Glick & Fiske, 1996). The ASI is a 22-item scale where participants indicate their agreement on a 6-point Likert-type scale (1 = “Completely disagree,” 6 = “Completely agree”) to various statements about men and women’s roles in society. A high score indicates higher levels of sexism, and a low score indicates lower levels of sexism. The ASI measures both HS and BS. The total scale had good reliability (α = .93), as did the sub-scales for BS (α = .96), and HS (α = .84).

##### Rape myth acceptance.

RMA was measured using the Acceptance of Modern Myths About Sexual Aggression (AMMSA; Gerger et al., 2007). The AMMSA consists of 30 statements reflecting common rape myths, and participants indicate their level of agreement on a 7-point Likert-type scale (1 = “Completely disagree,” 7 = “Completely agree”). A high score indicates higher levels of RMA. The scale had good reliability (α = .94).

##### Impression management.

Impression Management (IM) was measured using a shortened version of the Balanced Inventory of Desirable Responding (BIDR; Paulhus & Reid, 1991), namely, the BIDR-16 (Hart et al., 2015). The BIDR-16 measures the degree to which participants attempt to answer in socially desirable ways. The scale consists of 16 items, and participants indicate their agreement on a 7-point Likert-type scale (1 = “Strongly disagree,” 7 = “Strongly agree”). A high score indicates higher levels of impression management. This scale had good reliability (α = .79).

#### Outcome variables.

##### Victim culpability.

The degree to which participants implicated the victim was measured on a 12-item scale, which was specifically developed for this study. As proposed by Davies et al. (2001), items drew on blame (*n* = 4), responsibility (*n* = 3), and control (*n* = 5) as relevant constructs in victim precipitation and culpability. Some items were adapted from other research (Abrams et al., 2003; Ayala et al., 2018; Bieneck & Krahe, 2011; Davies & McCartney, 2003; Franklin & Garza, 2018). Participants indicated their agreement to various statements about the woman in the story on a 7-point Likert-type scale (1 = “Strongly disagree,” 7 = “Strongly agree”). A high score indicated that participants implicated the woman more in the assault. The scale had good reliability for all items (α = .88).

##### Perpetrator culpability.

The degree to which participants implicated the perpetrator was measured with the same items as for victim culpability (12 items) but replaced mentions of the woman in the story with the man. Participants indicated their agreement to various statements about the man in the story on a 7-point Likert-type scale (1 = “Strongly disagree,” 7 = “Strongly agree”). A high score indicated that participants implicated the man more in the assault. The scale had good reliability for all items (α = .89).

##### Demographic variables.

Participants were also asked to indicate their gender, sexuality, age, and ethnicity.

##### Attention checks.

To ensure participants engaged with the questions, a number of attention checks (Oppenheimer et al., 2009) were included within the questionnaire. Additionally, one manipulation check was asked. Those who failed more than one of the attention checks, or answered incorrectly to the manipulation check were excluded.

#### Procedure.

Data collection took place on Qualtrics Version XM (2020). After reading the study information, participants completed the AMMSA and the ASI. Then, they were presented with one of the possible four vignettes, which were randomized according to the block randomization option in Qualtrics (2020). After the vignette, participants completed the outcome measures which were all presented in a randomized order. Then, they completed IM and demographical measures. In total, the study took about 17 minutes.

#### Ethical considerations.

Ethical considerations were identical to Study One.

### Results

#### Data preparation.

In total, 262 participants completed the study. Participant who failed the attention checks (*n* = 7) were excluded. Then, negatively worded items were reverse scored, and new variables for total AMMSA, ASI, BIDR- 16, VB, and PB were created using the R package “PROscorerTools” (Baser, 2017). Each category of the victim–perpetrator relationship was dummy coded (0 = Not present, 1 = Present). The median absolute distribution (MAD) was used to identify outliers of the predictor variables which were greater than +/−3 MAD (Leys et al., 2013). Three outliers were identified and deleted. The final sample included 252 participants. There were no missing data.

Equivalence between conditions was assessed through a series of ANOVAs and Chi-square tests. There were no differences (*ps* > .05) between the conditions on any of the key variables: RMA, AS, IM, gender, and age.

#### Descriptive statistics.

Figure A2 (https://osf.io/42dwe/) illustrates levels of BS, HS, and RMA, comparing men and women. Figure A3 (https://osf.io/5zjwt/) illustrates victim and perpetrator culpability across rape conditions, comparing men and women, with means. Descriptive statistics can be found in Tables A2 and A3 (https://osf.io/92bq7/). Overall, level of victim culpability was low (*median* = 1.17, *min* = 1, *max* = 4.58), and overall perpetrator culpability was high (*median* = 7, *min* = 3.17, *max* = 7). As compared to women, men had higher levels of victim culpability, RMA, HS, and BS, but only RMA (*d* = .47, *p* = .002) and HS (*d* = .48, *p* = .002) remained significant when controlling for multiple comparisons (using the Bonferroni correction). There was no difference in perpetrator culpability.

#### Exploratory analyses.

Two repeated measures one-way ANOVAs were conducted to explore differences between the separate culpability sub-scales. The model for victim culpability was significant: *F*(2, 502) = 98.89, *p* < .0001. All pairwise comparisons were significant at *p* < .01. The largest difference was between victim control and victim blame (*p* < .0001, *d* = .95), where participants attributed significantly more control than blame to the victim across scenarios. The model for perpetrator culpability was not significant when controlling for multiple comparisons (*p* = .04).

#### Main analyses.

##### Bivariate correlations.

Correlation matrices were computed for all continuous variables, with Pearson’s R as the test statistic (Figure A4; https://osf.io/ex6sc/). All correlations were significant at *p* < .001. While victim and perpetrator culpability are negatively correlated, it is not a perfect correlation, suggesting that there is a utility in measuring these as separate constructs.

##### Victim–perpetrator relationship.

Two one-way ANOVAs were conducted to examine differences in victim and perpetrator culpability between the four relationship conditions. The model for victim culpability was significant: *F*(3, 248) = 4.77, *p* = .003, as was the model for perpetrator culpability: *F*(3, 248) = 3.90, *p* = .01. Bonferroni pairwise comparisons revealed that these effects were found between the acquaintance condition and the stranger home condition. Victim culpability was increased (*d* = .65, *p* = .002) and perpetrator culpability was decreased (*d* = .52, *p* = .006) in the acquaintance condition, as compared to the stranger home condition. The other comparisons were non-significant (*ps* > .05). Notably, there was no difference between the acquaintance condition and the stranger risk condition.

##### Regressions.

Regression plots with all predictor variables for victim and perpetrator culpability can be found in Figures A5 (https://osf.io/483s9/) and A6 (https://osf.io/ghtyk/). First, regressions for victim culpability were conducted (Table 1). In Model 1, victim–perpetrator relationship was entered, followed by BS, and interaction terms. HS, gender, and IM were all controlled for. Model 1 was significant, *F*(10, 239) = 10.15, adjusted *r*^2^ = .27, *p* < .0001. In Model 2, victim–perpetrator relationship was entered, followed by HS, and interaction terms. BS, gender, and IM were all controlled for. Model 2 was significant, *F*(10, 239) = 10.79, *r*^2^ = .28, *p* < .0001. In Model 3, victim–perpetrator relationship was entered, followed by RMA, and interaction terms. Gender, and IM were controlled for. Model 3 was significant, *F*(9, 240) = 11.50, adjusted *r*^2^ = .28, *p* < .0001. In these models, HS and RMA impacted victim culpability in all conditions but the partner condition, where victim culpability remained the same, regardless of participants’ sexist attitudes. In total, these models explained approximately 30% of the variance in victim culpability, with aggressively sexist factors being particularly important.

**Table 1.** Predictors for Victim and Perpetrator Culpability.

	Victim Culpability				Perpetrator Culpability			
	*b*	*se*	*t*	*p*	*b*	*se*	*t*	*p*
Victim–perpetrator relationship Partner Acquaintance Stranger risk Stranger home	–.61 –.07 –.14.14	.33 .30 .31 .31	−1.86 −.26 −.44.44	.06 .80 .66 .66	−.09 .27 .004 –.004	.26 .24 .25 .25	−.351 .12 .02− .02	.73 .26 .99 .99
Benevolent sexism	.04	.09	.50	.61	.03	.06	.44	.66
Hostile sexism	.26	.04	6.41	<.001***	−.12	.32	−3.74	<.001***
Rape myth acceptance	.34	.06	5.22	<.001***	–.11	.05	−2.1	.04^#^
Gender	.14	.07	1.85	.06	–.06	.06	−1.13	.26
Acquaintance*BS	–	–	–	–	–.17	.08	−2.03	.04^#^
Partner*BS	–.22	.11	−2.0	<.05^#^				
Acquaintance*HS	–	–	–	–	–.20	.06	−2.92	<.01**
Partner*HS	–.25	.09	−2.91	<.01**^a^	–	–	–	–
Stranger home*HS	–.17	.08	2.12	.03^#^	–	–	–	–
Partner*RMA	–.27	.10	−2.66	<.01*^a^	–	–	–	–
Acquaintance*RMA	–	–	–	–	–.20	.07	−2.78	<.01**

*Note*. ***p < .001, **p < .01, *p < .05.

^#^Not significant when controlling for multiple comparisons (Bonferroni).

^a^Significant as compared to all other victim–perpetrator conditions combined.

Second, regressions for perpetrator culpability were conducted (Table 1). In Model 1, victim–perpetrator relationship was entered, followed by BS, and interaction terms. HS, gender, and IM were all controlled for. Model 1 was significant, *F*(10, 239) = 4.9, adjusted *r*^2^ = .14, *p* < .0001. In Model 2, victim–perpetrator relationship was entered, followed by HS, and interaction terms. BS, gender, and IM were all controlled for. Model 2 was significant, *F* (10, 241) = 5.77, adjusted *r*^2^ = .16, *p* < .0001. In Model 3, victim–perpetrator relationship was entered, followed by RMA, and interaction terms. Gender, and IM were controlled for. Model 3 was significant, *F*(10, 240) = 6.76, adjusted *r*^2^ = .17, *p* < .0001. Similar to analyses for victim culpability, HS had the strongest effects on its own, which was qualified by an interaction between hostile sexist attitudes (HS and RMA) and the acquaintance condition, where perpetrator culpability decreased in line with the strength of these attitudes, as compared to all other conditions. In total, these models explained around 17% of perpetrator culpability. It is notable that gender was not a significant predictor in any of the models for victim and perpetrator culpability. However, it is likely that this is accounted for by men’s higher levels of HS and RMA.

### Discussion

Study Two established that it is aggressively sexist attitudes, not benevolently sexist attitudes, that increase victim culpability, and reduce perpetrator culpability. Importantly, findings showed that the difference in victim and perpetrator culpability was found between the acquaintance and stranger home condition and that levels of victim and perpetrator culpability were similar between the stranger risk condition and the conditions where the victim knew the perpetrator. This is in direct contrast with Abrams et al. (2003), who found an effect of the victim–perpetrator relationship, despite the stranger rape victim being portrayed as walking home alone. As previously discussed, this highlights the need to standardize manipulations across vignette conditions, as not all women raped by a stranger will be blamed less than women who know the perpetrator. This, therefore, calls into question some of the past results in the area of victim–perpetrator relationship and victim blame where variations of stranger rape vignettes have been used indiscriminately. Moreover, the woman subjected to rape was generally ascribed more control than blame, suggesting that while participants may consider that the overall assault should not be blamed on the woman, they still perceive the woman to have had some control over the outcome, thus indirectly suggesting the woman to have behaved in a way which caused the outcome. This suggests that future research needs to be clear on which construct is being measured, as this will likely affect results.

Finally, RMA and HS increased victim culpability in all conditions other than the partner condition, which remained relatively unaffected by aggressively sexist attitudes. Relatedly, RMA and HS mainly decreased perpetrator culpability in the acquaintance condition. These results suggest that sexist attitudes might extend a level of protection to intimate partners, possibly due to their associations with traditional notions of intimate heterosexual relationships (e.g., men as being the protectors in relationships), while exonerating men for raping casual acquaintances, to whom this protection presumably does not extend (Glick & Fiske, 1996; Masser et al., 2006).

## Study Three

Study Two established the importance of aggressively sexist attitudes in ratings of victim and perpetrator culpability. While attitudes towards the woman subjected to rape, as well as the perpetrator, can have severe implications after the assault (Gerger et al., 2007; Smith, 2018), sexist attitudes have also been proposed to impact men’s tendency to sexually aggress in the first place (Masser et al., 2006). As established above (and in much prior research; Grubb & Turner, 2012), men tend to score higher on measures on aggressive sexism. These attitudes are primarily concerned with negative evaluations of women more generally, and the minimization of the severity of sexual violence, which links to a greater propensity towards sexual violence among men (Masser et al., 2006). As men are more likely (ONS, 2019) to assault women they know (and have a greater likelihood of being acquitted of this crime), it is anticipated this will be particularly pronounced in scenarios where the woman subjected to rape knows the perpetrator. The final study, therefore, aims to examine the relationship between victim–perpetrator relationship and rape proclivity, and how this is moderated by HS and BS, and RMA.

### Methods

#### Design.

This study utilized a between-subjects design, and included three vignettes (vignettes A, C, and E) as validated in Study One, varying levels of familiarity between stranger, acquaintance, and partner. Predictor variables were BS, HS, and RMA. The outcome variable was rape proclivity.

#### Data analysis strategy.

Data analysis strategy was identical to that of Study Two.

#### Participants.

Sample size was based on a power calculation conducted in R (R Core Team, 2020), using the “pwr” package (Champely, 2020). The power level was set at 95% with six predictor variables, and the target effect size was small-medium (*f*^2^ = .10), as based on previous research in this area (Abrams et al., 2003). Significance level was set at *p* < .05. As illustrated in Figure A1 (https://osf.io/xdavj/), 177 participants were needed. One-hundred and eighty-two heterosexual men were included in the final analysis. Participants were UK residents with a mean age of 34.98 years (*SD* = 11.54, *median* = 32, *min* = 18, *max* = 82). In terms of ethnicity, participants were White (88%), Asian (6.6%), Black British (2.8%), and of Other or Mixed ethnicity (2.6%). Participants were paid £1.46 for taking part.

#### Materials.

Materials were identical to those of Study Two, apart from the removal of the stranger risk condition (vignette F), and the outcome variable being rape proclivity, rather than victim and perpetrator culpability.

##### Vignettes.

Participants were presented with one of three vignettes, depicting a partner rape (A), an acquaintance rape (C), or a stranger rape with the victim at home (E). Further vignette details can be found in Study One.

##### Predictor variables.

*Ambivalent sexism.* AS was measured using the ASI (Glick & Fiske, 1996). The total scale had good reliability (α = .90), as did the sub-scales for BS (α = .83), and HS (α = .91).

*Rape myth acceptance.* This was measured using the AMMSA (Gerger et al., 2007). The scale had good reliability (α = .91).

*Impression management.* IM was measured using a shortened version of the BIDR (Paulhus & Reid, 1991), the BIDR − 16 (Hart et al., 2015), which had good reliability (α = .79).

*Rape proclivity* (RP). The likelihood of engaging in sexual aggression was measured on an 8-item, 5-point Likert-type scale, which was developed specifically for this study. Some items were adapted from previous research in this area (Abrams et al., 2003; Chiroro et al., 2004; Masser et al., 2006). An important difference between our items and those previously used was that all questions were as subtle as possible in nature, for example, “Would you have behaved like the man in the story?” was changed to “Is it possible you might have behaved like the man in the story?” Items tapped into sexual arousal (*n* = 3), likelihood of engaging in sexual violence (*n* = 3), and the belief that women enjoy sexual domination (*n* = 2). A higher score indicated a higher acceptance of interpersonal aggression, and likelihood of engaging in sexual violence. The scale had good reliability (α = .85).

*Demographic variables.* Participants were also asked about their age and ethnicity.

*Attention check.* These measures were identical to Study Two.

#### Procedure and ethical considerations.

Procedure and ethical considerations were identical to Study Two.

### Results

#### Data preparation.

In total, 188 participants completed the study. Participants who failed the attention checks (*n* = 4) were excluded. Then, negatively worded items were reverse scored, and new variables for total AMMSA, ASI, BIDR-16, and RP were created (Baser, 2017). Each category of the victim–perpetrator relationship was dummy coded (0 = Not present; 1 = Present). The MAD was used to identify outliers of the predictor variables which were greater than +/−3 MAD (Leys et al., 2013). Two outliers were deleted. The final sample included 182 participants. There were no missing data.

#### Equivalence between groups.

Equivalence between conditions was assessed through a series of ANOVAs and Chi-square tests. There were no differences (*ps* > .05) between the conditions on any of the key variables: RMA, AS, IM, and age.

#### Descriptive statistics.

Figure A7 (https://osf.io/d4ye3/) illustrates levels of BS, HS, and RMA. Figure A8 (https://osf.io/fbh6p/) illustrates rape proclivity across conditions. Table A4 (https://osf.io/92bq7/) provides an overview of means and SDs for all these values. The overall level of rape proclivity was low (*M* = 1.50, *SD* = 0.58, *min* = 1, *max* = 4.25).

#### Main analyses.

##### Bivariate correlations.

Correlation matrices were computed for all continuous variables, with Pearson’s R as the test statistic. These correlations can be found in Figure A9 (https://osf.io/9kdhe/). All correlations were significant at *p* < .001, apart from BS and rape proclivity, which was non-significant when controlling for multiple comparisons.

##### Victim–perpetrator relationship.

A one-way ANOVA was carried out to examine the difference in rape proclivity between the three relationship conditions. The model was significant: *F*(2, 129) = 7.24, *p* = .001. Bonferroni pairwise comparisons revealed that these effects were found between the known-perpetrator conditions and the stranger condition. Compared to the stranger condition, rape proclivity was increased in the partner condition (*d* = .61, *p* = .006), and in the acquaintance condition (*d* = .64, *p* = .002).

##### Regressions.

Regression plots with all predictor variables for rape proclivity can be found in Figure A10 (https://osf.io/anvur/), and detailed results in Table 2. In Model 1, victim–perpetrator relationship was entered, followed by BS, and interaction terms. HS and IM were controlled for. Model 1 was significant, *F*(7, 174) = 6, adjusted *r*^2^ = .16, *p* < .0001. In Model 2, victim–perpetrator relationship was entered, followed by HS, and interaction terms. BS and IM were controlled for. Model 2 was significant, *F*(7, 174) = 5.90, *r*^2^ = .16, *p* < .0001. In Model 3, victim–perpetrator relationship was entered, followed by RMA, and interaction terms. IM was controlled for. Model 3 was significant, *F*(6, 175) = 13.16, adjusted *r*^2^ = .30, *p* < .0001. Together, these models demonstrate that aggressively sexist attitudes (HS and RMA) have the most considerable effects on rape proclivity, with no interaction effects. The model with RMA and victim–perpetrator relationship explained the most variance, which was approximately 30%.

**Table 2.** Predictors for Rape Proclivity.

	Rape Proclivity			
	*b*	*se*	*t*	*p*
Victim–perpetrator relationship Partner Acquaintance Stranger home	.10.03 –.10	.40 .39 .39	.25 .07 −.25	.80 .94 .80
Benevolent sexism	.43	.33	1.27	.21
Hostile sexism	.15	.5	3.34	<.01**
Rape myth acceptance	.95	.32	−.60	<.01**
Impression management	–.01	.05	2.91	.02^#^

*Note*. **p < .01.

^#^Not significant when controlling for multiple comparisons (Bonferroni).

### Discussion

Rape proclivity was significantly higher in all conditions where the perpetrator knew the woman, with a slightly larger effect as measured by Cohen’s *d* for the acquaintance condition. It, therefore, appears that while people generally are the most likely to exonerate the perpetrator of acquaintance rape (particularly if they are high in aggressively sexist attitudes), the tendency for sexual violence is equal in broader known-perpetrator scenarios. As such, this warrants future research into the potential differences in how men perceive perpetrators more generally, and how they may judge their own potential actions. As expected, aggressively sexist attitudes predicted greater rape proclivity. However, there was no interaction effect in any of the conditions.

## General Discussion

Through a series of related studies, this research produced a number of validated vignettes depicting rape with varying levels of victim–perpetrator relationships, which were experimentally implemented together with measures of AS and RMA to examine culpability ratings and rape proclivity. In doing so, this research has developed valid and believable material relating to rape, which can be reliably used in future research in this area, thus filling a considerable gap in past research. For instance, these vignettes could be used to examine attitudes to rape among public, as well as professional samples; it would be particularly relevant to examine groups that regularly come into contact with women who have been subjected to rape (e.g., medical professionals; counselors, etc.). Long-term, this material could also be utilized to evaluate the effectiveness of interventions developed to change attitudes and beliefs about rape, which could have important implications for policy and practice.

This research also established that attributions in rape cases appear to be mainly influenced by aggressively sexist attitudes. Specifically, women who are raped by someone they know are considered the most culpable (particularly in terms of control), which suggests that there may be a utility for future research to further refine measures of victim blame. Relatedly, men score higher on aggressively sexist attitudes than women, which may link to their rape proclivity in known-perpetrator scenarios. These findings are broadly in line with past research, but also present some relevant differences to what has been previously found. The effect of the victim–perpetrator relationship on victim blame is well-established (Persson & Dhingra, 2020), but contrary to some past research (Bieneck & Krahe, 2011), this study did not find a linear relationship between relationship-proximity and outcome variables, which is in line with findings from our previous meta-analysis. Further, we propose that the standardization of flirtatious behavior contributed to the victim being blamed less for the partner rape, as past research has often included this in the date rape condition (e.g., Bridges, 1991). Further, as variations in the stranger rape condition produced different results, we also highlight the need for future research to consistently clarify how this condition is operationalized, and we also recommend that this is pre-registered. Reproducible research practices may, therefore, have positive implications for research in this area.

Past research using similar methods has established a robust link between aggressively sexist attitudes and blame attributions and rape proclivity (Abrams et al., 2003; Franklin & Garza, 2018; Persson et al., 2018), and the current research largely reproduces these findings among well-powered community samples, using a robust methodology. In the context of the current findings, aggressively sexist attitudes may particularly contribute to the belief that the woman had some control over the outcome of the rape, which is a novel contribution to the literature. Through this, these attitudes also link with rape proclivity, as they serve to justify rape through the woman’s behavior and allow the perpetrator to minimize his actions. Linking with past research, this is particularly salient in scenarios where the perpetrator can view his actions as more justifiable and less malevolent (Masser et al., 2006), that is, in scenarios of knowing the victim. This was evidenced in Study Three, which found the highest levels of rape proclivity in the acquaintance and partner rape scenarios. A central tenet of sexist attitudes is viewing men as protectors of their partner in relationships (protective paternalism; Glick & Fiske, 1996), but it is possible that various victim-blaming strategies allow men to disregard this aspect of BS when making judgments about rape cases. Further research would, therefore, benefit from exploring the limits of protective paternalism in the context of partner rape further, and it would also be relevant to directly investigate whether men hold themselves to different standards than they hold other men. Finally, a considerable strength of the results of the current study is the confidence with which the manipulations can be considered to have impacted the outcome variables. Unlike much previous research (Persson & Dhingra, 2020), Study One established that participants identified all key manipulations, and these materials can, therefore, be used to produce evidence-based research with relevance for policy and practice, in the context of improving provisions for women who have been subjected to rape.

### Limitations and Future Directions

While the current research aimed to account for limitations in past research, there are a number of points to highlight regarding directions for future research. The sample for this research was drawn exclusively from the United Kingdom and was overwhelmingly White. Therefore, the extent to which findings can be applied to other countries and contexts are, currently, limited. A relevant extension to this study would be to implement these methods somewhere with (even) more permissive attitudes towards sexual violence in relationships. We would, therefore, welcome the re-use of these vignettes and measures in other contexts. As women from ethnic minorities are particularly susceptible to victim blame and poor police responses (Smith, 2018), future research would benefit from including a more diverse sample. While the outcome variables have been drawn from past research and had good reliability, results should also be interpreted in the context of these measures being relatively novel, which may impact validity. It is also possible that some items tapped into more than one of the culpability domains.

Moreover, as noted by Smith (2018), rape myths and aggressively sexist attitudes are particularly resistant to interventions on an individual level, as rape myths are not about ignorance. Rather, rape myths are the logic with which truth is established, and are part of the cultural scaffolding which genders the notion of truth, and holds women to a hypothetical ideal which all behavior is measured against, as drawing on MacKinnon’s notion of the “legally perfect rape” (1989). This, therefore, suggests that the way to counteract these attitudes may not lie in interventions on an individual level, as they will have limited effectiveness (Wright et al., 2018). Rather, this would suggest that aggressively sexist attitudes will need to be targeted on a broader level, through re-defining the general cultural and legal landscape, which may in some instanced perpetuate present inequalities (Kennedy, 2011; MacKinnon, 1989; Smith, 2018). This further suggests that an adversarial legal system may not be in the best interest of women subjected to rape, indicating that future research may wish to investigate whether an inquisitorial system would be more suitable for the needs of women. Finally, the role of social media in perpetuating rape myths and victim blame may be a particularly relevant avenue for future research (Stubbs-Richardson et al., 2018). While this study did not find that general sexism (e.g., BS) was directly related to culpability and rape proclivity, it does nonetheless contribute to the cultural landscape where aggressively sexist attitudes are legitimized and maintained (Chapleau et al., 2007).

### Conclusion

This research provides a timely update to previous findings on the victim–perpetrator relationship and attributions in rape cases. Through three separate studies, this research found that aggressively sexist attitudes are associated with lower perpetrator culpability, increased victim culpability, as well as greater rape proclivity. It further found that victims of acquaintance rape are considered the most culpable for the rape and that men report the highest level of rape proclivity in a known-perpetrator scenario. While BS did not impact directly on any of the measures, we propose that its indirect legitimization of inequality and aggressive attitudes towards women scaffolds both HS and RMA. By applying a reproducible methodology, including validated vignettes that were considered both believable and realistic by participants, these findings provide a robust evidence base from which to draw conclusions, as well as materials that can be reliably used in future research in this area (e.g., in research into determinants, correlates, and outcomes of victim blame). Long-term, this can contribute to the development of broad, long-term interventions to change public attitudes to rape (in the context of also targeting general sexism), and does, as such, make a significant contribution to knowledge in this area, particularly in the context of policy and practice.

## Supplemental Material

Supplemental material for this article is available online. Click here for additional data file. Supplemental Material for Moderating Factors in Culpability Ratings and Rape Proclivity in Stranger and Acquaintance Rape: Validation of Rape Vignettes in a Community Sample by Sofia Persson and Katie Dhingra, in Journal of Interpersonal Violence

## Author Biographies

**Sofia Persson**, PhD, is a lecturer in Psychology at Leeds Beckett University, United Kingdom. Her research focuses on blame attributions in rape cases, and how this is influenced by Rape Myth Acceptance, Ambivalent Sexism, and the victim–perpetrator relationship. She is an Open Science advocate, and the program lead for Open Research and Scholarship within the Centre for Psychological Research at Leeds Beckett University.

**Katie Dhingra**, PhD, is a reader/associate professor in Psychology at Leeds Beckett University, United Kingdom. Her research primarily focuses on self-harm (both suicidal behavior and non-suicidal self-injury), violence against women and children, and psychological responses to traumatic life events.
