# Supplementary material for: Moderating Factors in Culpability Ratings and Rape Proclivity in Stranger and Acquaintance Rape: Validation of Rape Vignettes in a Community Sample
Source: J Interpers Violence. 2021 Feb 8;37(13-14):NP11358–85. doi: 10.1177/0886260521991294 (PMC9253925; doi:10.1177/0886260521991294)
Supplement: Supplemental material for this article is available online. [file sj-pdf-1-JIV-10.1177_0886260521991294.pdf]

APPENDIX A

Manipulation Check and Credibility Measures for Study One

Measures for Partner Conditions

Manipulation Checks

1) Based on the information above, was this sexual encounter non-consensual? (Yes/no)

2) Based on the information above, was Joseph a stranger to Amy? (Yes/no)

3) Based on the information above, was Joseph Amy's boyfriend? (Yes/no)

4) Based on the information above, did Amy engage in any flirtatious behaviour before Joseph had sex with her? (Yes/no)

Credibility Measures

1) Do you think the account you just read is believable? If no, please tell us why. (Yes/no)

2) Do you think the account you just read is realistic? If no, please tell us why. (Yes/no)

3) Do you think you could make similar judgements about the account you just read, as you would do about a real-life situation, if you had the same amount of information? If no, please tell us why. (Yes/no)

Measures for Stranger Conditions

Manipulation Checks

1) Based on the information above, was this sexual encounter non-consensual? (Yes/no)

2) Based on the information above, was the man a stranger to Hannah? (Yes/no)

3) Based on the information above, did Hannah know the man? (Yes/no)

4) Based on the information above, did Hannah engage in any flirtatious behaviour before the man had sex with her? (Yes/no)

Credibility Measures

1) Do you think the account you just read is believable? If no, please tell us why. (Yes/no)

2) Do you think the account you just read is realistic? If no, please tell us why. (Yes/no)

3) Do you think you could make similar judgements about the account you just read, as you would do about a real-life situation, if you had the same amount of information? If no, please tell us why. (Yes/no)

Measures for Acquaintance conditions

Manipulation Checks

1) Based on the information above, was this sexual encounter non-consensual? (Yes/no)

2) Based on the information above, was Nick a stranger to Chaya? (Yes/no)

3) Based on the information above, did Chaya know Nick? (Yes/no)

4) Based on the information above, did Chaya engage in any flirtatious behaviour before Nick had sex with her? (Yes/no)

Credibility Measures

1) Do you think the account you just read is believable? If no, please tell us why. (Yes/no)

2) Do you think the account you just read is realistic? If no, please tell us why. (Yes/no)

3) Do you think you could make similar judgements about the account you just read, as you would do about a real-life situation, if you had the same amount of information? If no, please tell us why. (Yes/no)

Attention Check Item (presented last, for all participants)

1) In the final story you read, did the woman order a take-away? (Yes/no)

Manipulation and Attention Checks for Study Two

1) (item presented within the AMMSA).

It can be difficult to answer many questions about sexual relationships when you are part of a study. Select "strongly agree" here.

2) (item presented within the ASI).

You should select "disagree somewhat" here.

3) (item presented within the victim blame scale [Study 2] or the rape proclivity scale [Study 3]).

The woman is a recruitment platform. Please select "strongly disagree"

4) (manipulation check; immediately following the vignette).

In the story you just read, did anyone order a take-away? (Yes/no)

APPENDIX B

Vignettes used in Studies One, Two, and Three

A. **Partner – no flirtation**

Amy is at home with her boyfriend Joseph, watching TV. They have had a nice evening, eating a meal that they cooked together. It is getting late, and Amy wants to get ready to go to bed – she has an early start at work tomorrow. As she is about to get up from the sofa, Joseph starts to kiss her. Amy says she is too tired, and continues to get up off the sofa. Joseph pulls her back down on the sofa, and starts pulling off her clothes, telling her how attractive she is. Amy says no, but Joseph continues anyway. Despite Amy's repeated objections Joseph holds her down and has sex with her.

B. **Partner - flirtation**

Amy is at home with her boyfriend Joseph, watching TV. They have had a nice evening, eating a meal that they cooked together. It is getting late, and Amy wants to get ready to go to bed – she has an early start at work tomorrow. As she is about to get up from the sofa, Joseph starts to kiss her. Amy kisses him back and lies on top of him. After a little while, Amy says she is too tired, and continues to get up off the sofa. Joseph pulls her back down on the sofa, and starts pulling off her clothes, telling her how attractive she is. Amy says no, but Joseph continues anyway. Despite Amy's repeated objections Joseph holds her down and has sex with her.

C. **Acquaintance – no flirtation**

Chaya is at home with her friend Nick, watching TV. They have had a nice evening, eating a meal that they cooked together. It is getting late, and Chaya wants to get ready to go to bed –

she has an early start at work tomorrow. As she is about to get up from the sofa, Nick starts to kiss her. Chaya says she is too tired, and continues to get up off the sofa. Nick pulls her back down on the sofa, and starts pulling off her clothes, telling her how attractive she is. Chaya says no, but Nick continues anyway. Despite Chaya's repeated objections, Nick holds her down and has sex with her.

D. **Acquaintance – flirtation**

Chaya is at home with her friend Nick, watching TV. They have had a nice evening, eating a meal that they cooked together. It is getting late, and Chaya wants to get ready to go to bed – she has an early start at work tomorrow. As she is about to get up from the sofa, Nick starts to kiss her. Chaya kisses him back and lies on top of him. After a little while, Chaya says she is too tired, and continues to get up off the sofa. Nick pulls her back down on the sofa, and starts pulling off her clothes, telling her how attractive she is. Chaya says no, but Nick continues anyway. Despite Chaya's repeated objections, Nick holds her down and has sex with her.

E. **Stranger Home**

Hannah is at home alone, watching TV. She has had a nice evening, eating a meal that she cooked herself. It is getting late, and Hannah wants to get ready to go to bed – she has an early start at work tomorrow. As she is about to get up off the sofa, she hears a sound coming from the door. A man whom she has never seen before has forced his way into the flat, and quickly approaches her. He pulls her down on the sofa, and starts pulling off her clothes, telling her how attractive she is. Hannah says no, but the man continues anyway. Despite Hannah's repeated objections, the man holds her down and has sex with her.

F. **Stranger Risk**

Hannah is walking home alone. She has had a nice evening, eating a meal in a restaurant with friends. It is getting late, and she is ready to get home and go to bed – she has an early start at work tomorrow. As she is approaching her flat, a man she has never seen before approaches her from behind. Hannah tries to walk away, but the man pulls her down on the ground, and starts pulling off her clothes, telling her how attractive she is. Hannah says no, but the man continues anyway. Despite Hannah's repeated objections, the man holds her down and has sex with her.

APPENDIX C

Tables for Study One

Table A1

Manipulation Check and Believability for all Vignettes

		MC1	MC2	MC3	MC4	C1	C2	C3
Vignette A								
	Yes	98.39(61)	0(0)	100(62)	3.23(2)	90.32(56)	93.55(58)	91.94(57)
	No	1.51(1)	100(62)	0(0)	96.77(60)	9.68 (8)	6.54(4)	8.06(5)
Vignette B								
	Yes	94.03 (63)	0(0)	100(67)	70.15(47)	97.01(65)	94.03(63)	91.04(61)
	No	6.85(5)	100(67)	0(0)	29.85(20)	2.99(2)	5.97(4)	8.96(6)
Vignette C								
	Yes	100(68)	0(0)	100(68)	1.47(1)	92.65(63)	92.65(63)	100(68)
	No	0(0)	100(68)	0(0)	98.53(67)	7.35(5)	7.35(5)	0(0)
Vignette D								
	Yes	96.72(59)	0(0)	100(61)	88.52(54)	98.36(60)	98.36(60)	91.80(56)
	No	3.28(2)	100(61)	0(0)	11.48(7)	1.64(1)	1.64(1)	8.20(5)
Vignette E								
	Yes	98.46 (64)	100(65)	0(0)	100(65)	90.77(59)	84.62(55)	100(65)
	No	1.54(1)	0(0)	100(65)	0(0)	9.23(6)	15.38(10)	0(0)
Vignette F								
	Yes	98.44(63)	98.44(63)	0(0)	100(64)	96.88(62)	98.44(63)	98.44(63)
	No	1.56(1)	1.56(1)	100(64)	0(0)	3.12(2)	1.56(1)	1.56(1)

Note.

MC1 = “Was this non-consensual?” ; MC2 = “Was the man a stranger to the woman?”; MC3 = “Was the man the woman’s friend/boyfriend?”; MC4 = “Did the woman engage in any flirtatious behaviour before the man had sex with her?”; C1 = “Do you think the account you just read is believable?”; C2 = “Do you think the account you just read is realistic?”; C3 = “Do you think you could make similar judgements about the account you just read, as you would do a real-life situation?”.

APPENDIX D

Victim and Perpetrator Culpability Measures

These measures have been used to measure culpability in response to stranger and acquaintance rape. Questions were answered on a 7-point Likert-type scale (1 = "strongly disagree", 7 = "strongly agree").

Scoring:

Blame: items 1, 3, 6, 10

Responsibility: items 8, 11, 12

Control: items 2, 4, 5, 7, 9

Rev-scored: items 1, 5, 8

Victim Culpability

Instructions: On the next page you will be asked a series of questions about your opinions of the woman in the story you just read. There are no right or wrong answers, please just give your honest opinion.

1) The woman in this story cannot be blamed for what happened.

2) The woman in this story could have avoided the incident.

3) This incident was the woman's fault.

4) The woman had control over the situation.

5) The woman cannot be blamed for what happened to her. She was just in the wrong place at the wrong time.

6) The woman's behaviour was to blame for what the man did.

7) The woman could have acted differently to avoid this outcome.

8) The woman is not responsible for these circumstances.

9) The situation was influenced by the woman.

10) The woman is to blame for these circumstances.

11) The woman is responsible for what happened.

12) The woman is the cause of what happened.

Perpetrator Culpability

Instructions: On the next page you will be asked a series of questions about your opinions of the man in the story you just read. There are no right or wrong answers, please just give your honest opinion.

1) The man in this story cannot be blamed for what happened.

2) The man in this story could have avoided the incident.

3) This incident was the man's fault.

4) The man had control over the situation.

5) The man cannot be blamed for what happened to the woman. He was just in the wrong place at the wrong time.

6) The man's behaviour was to blame for what he did.

7) The man could have acted differently to avoid this outcome.

8) The man is not responsible for these circumstances.

9) The situation was influenced by the man.

10) The man is to blame for these circumstances.

11) The man is responsible for what happened.

12) The man is the cause of what happened.

APPENDIX E

Figures for Study Two

Figure A1

Sample Size Calculation Plot

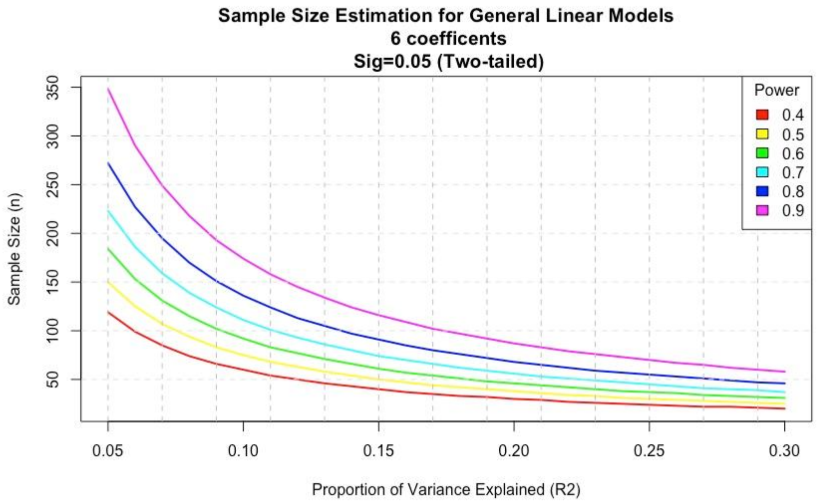

Figure A2

Scores on all Predictor Variables Separated by Gender

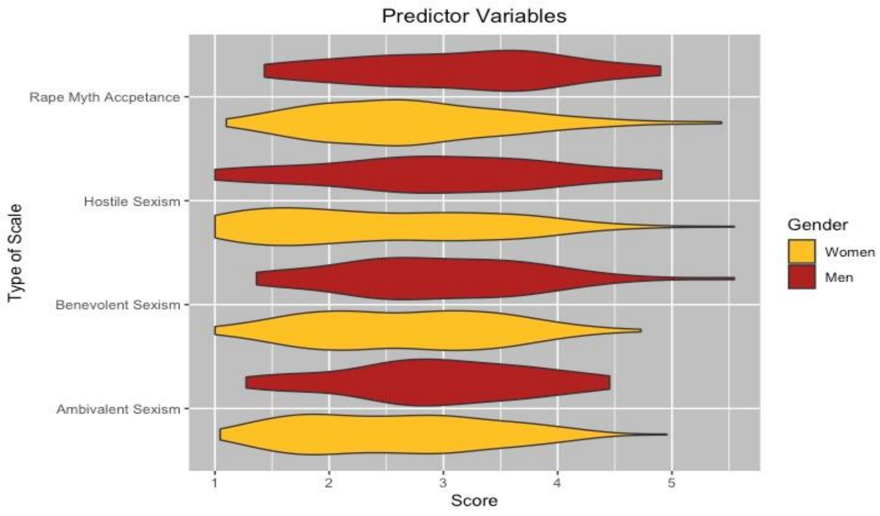

Figure A3

Victim-Perpetrator Relationship and Culpability Separated by Gender (with Means)

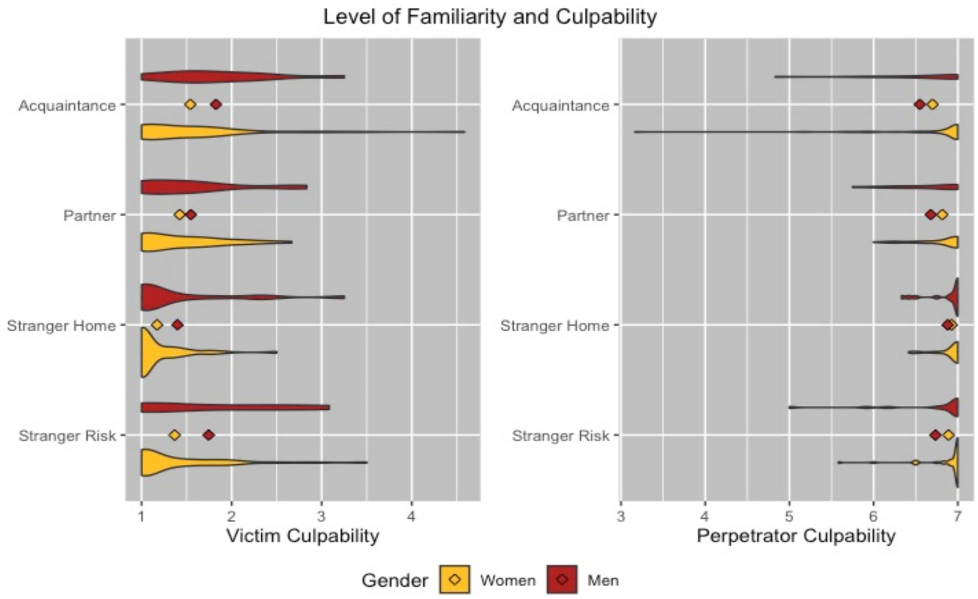

Figure A4

Bivariate Correlations with Correlation Coefficients

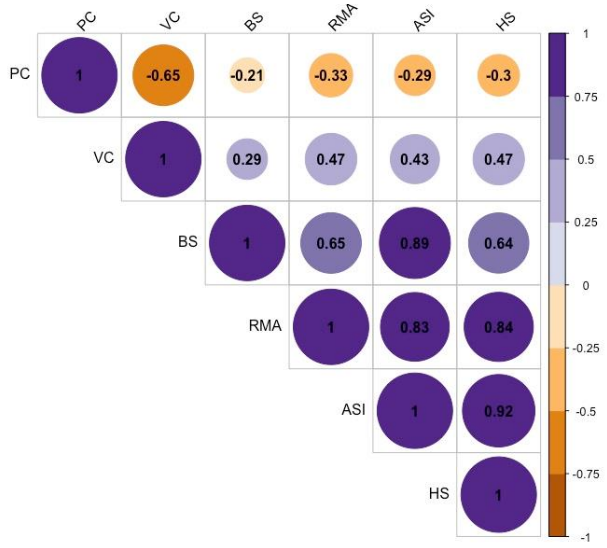

Figure A5

Regression Plots for all Predictor Variables and Victim Culpability as Separated by Victim-Perpetrator-Relationship

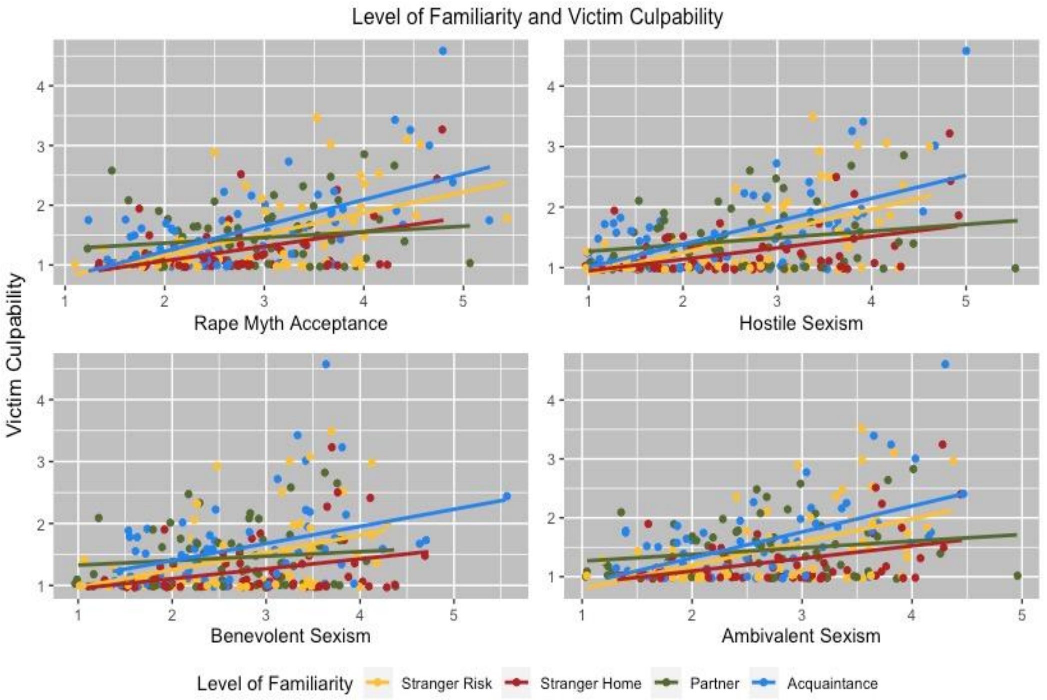

Figure A6

Regression Plots for all Predictor Variables and Perpetrator Culpability as Separated by Victim-Perpetrator-Relationship

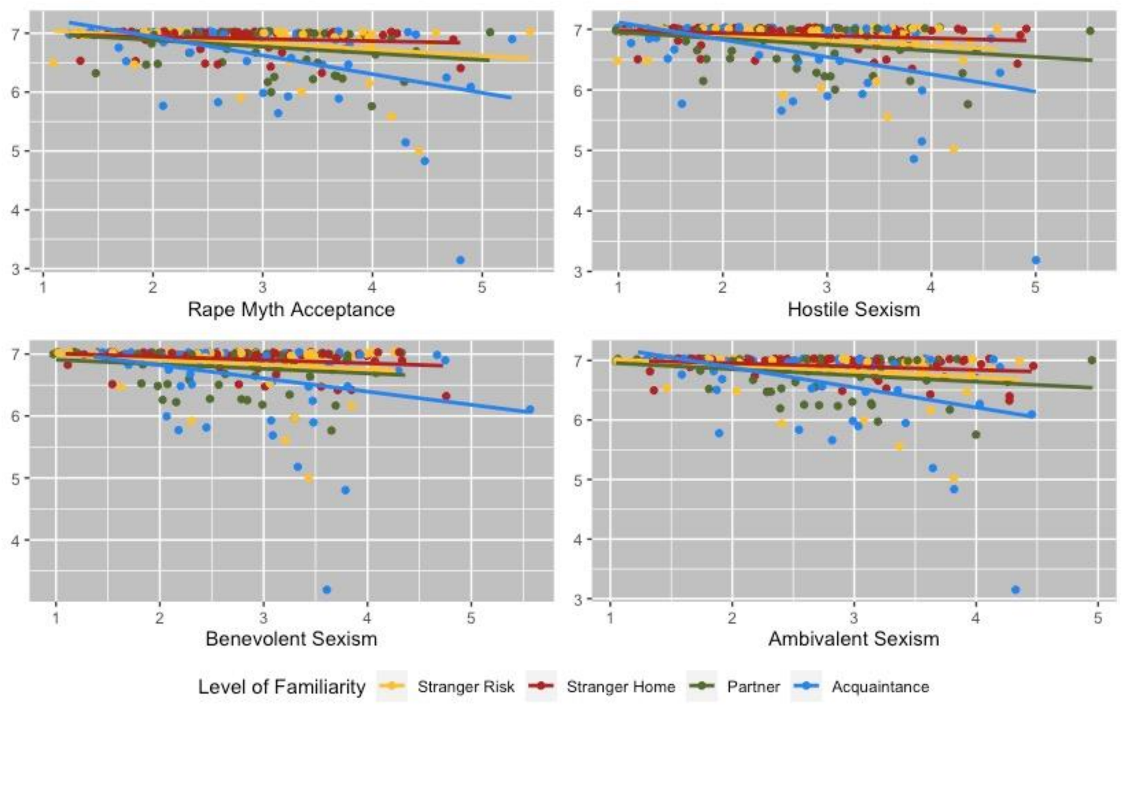

APPENDIX F

Tables for Study Two

Table A2

Means and Standard Deviations across all Variables

	N	VC	PC	BS	HS	RMA
Victim-perpetrator relationship						
Partner	63	1.45(0.48)	6.79 (0.32)	-	-	-
Acquaintance	63	1.60(0.68)	6.67 (0.66)	-	-	-
Stranger Home	65	1.23(0.43)	6.92(0.18)	-	-	-
Stranger Risk	61	1.47(0.66)	6.85(0.38)	-	-	-
Gender						
Women	189	1.38(0.54)’	6.83 (0.41)	2.67 (0.84)’	2.46 (1.01)**	2.72 (0.87)**
Men	62	1.61 (0.48)	6.72 (0.48)	2.95(0.87)	2.94 (1)	3.13(0.90)
Non-Binary	2	1.04(0.06)	7(0)	1.35 (0.51)	1.09(0)	1.53(0.19)
Total	252	1.43(0.58)	6.81 (0.43)	2.73(0.86)	2.57(1.03)	2.81(0.90)

Note. Value lower than compared to men, and significant as follows:
***p < .001, **p < .01, * p < .05m ‘Not significant when controlling for multiple comparisons (Bonferroni)

Table A3

Means and Standard Deviations across the Culpability Subscales

	Victim Culpability	Perpetrator Culpability
Blame	1.20 (0.46)	6.82 (0.52)
Responsibility	1.28(0.60)	6.83 (0.50)
Control	1.71(0.87)	6.78 (0.44)

Appendix G

Rape Proclivity Scale

These measures have been used to measure rape proclivity in response to stranger and acquaintance rape. Questions were answered on a 5-point Likert-type scale (1 = “strongly disagree”/” definitely not”, 7 = “strongly agree”/” definitely yes”).

Scoring:

Sexual arousal: items 1, 3, 8

Propensity to engage in sexual violence: items 2, 6, 7

Women enjoy domination: items 4, 5

Rev-scored: item 7

Instructions: Below are some questions about your personal opinions on the story you just read. There are no right or wrong answers, please just give your honest opinion. All your responses are anonymous and confidential.

1) Is it possible you might have felt sexually aroused as the man in this story?

2) Is it possible you could have behaved like the man in the situation just described, if you knew for certain no one would find out?

3) Is it possible you could have somewhat enjoyed getting your way like the man in this situation?

4) Do you agree that in sexual encounters women like to resist, but might eventually enjoy it?

5) How likely is it that the woman in this story eventually enjoyed the sexual activity?

6) Might you have behaved like the man if you knew for certain no one would find out?

7) Even if you knew no one would find out, would you have avoided acting like the man in the described situation?

8) Might you have enjoyed acting like the man in the situation you just read about?

Appendix H

Figure A7

Victim-Perpetrator Relationship and Rape Proclivity

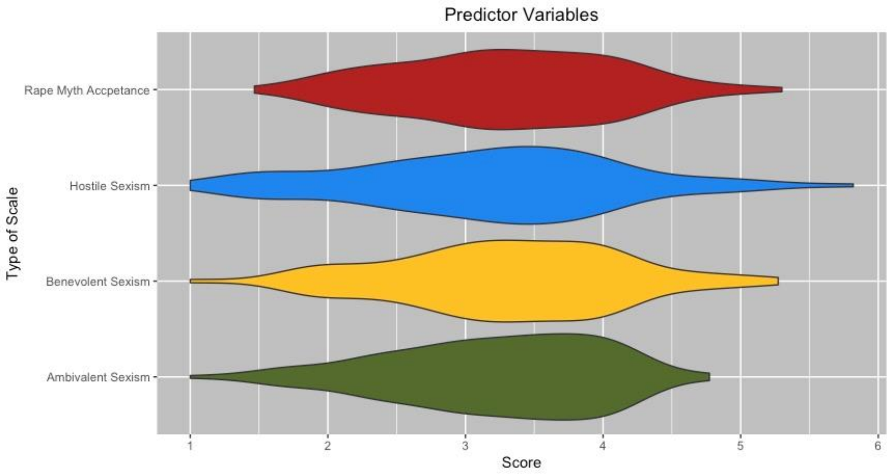

Figure A8

Scores on all Predictor Variables

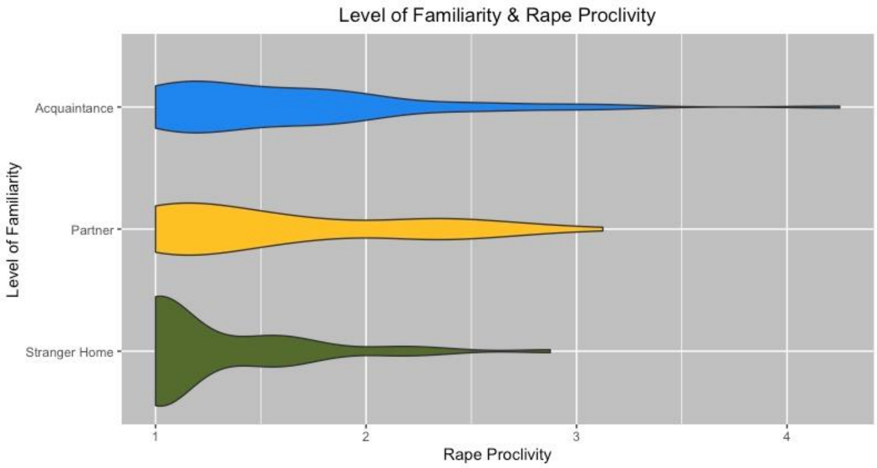

Figure A9

Bivariate Correlations with Correlation Coefficients

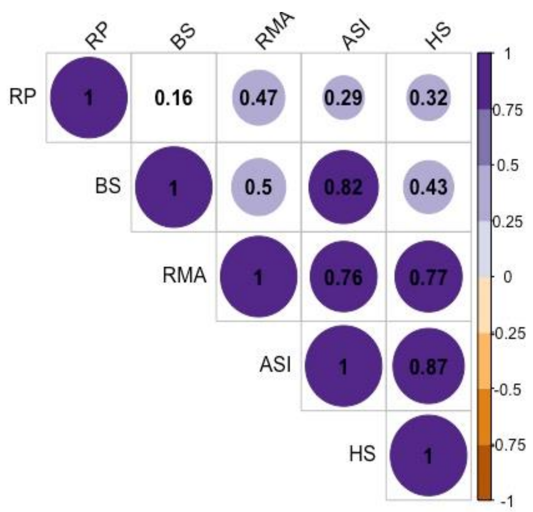

Figure A10

Regression Plots for all Predictor Variables on Rape Proclivity as Separated by Victim-Perpetrator-Relationship

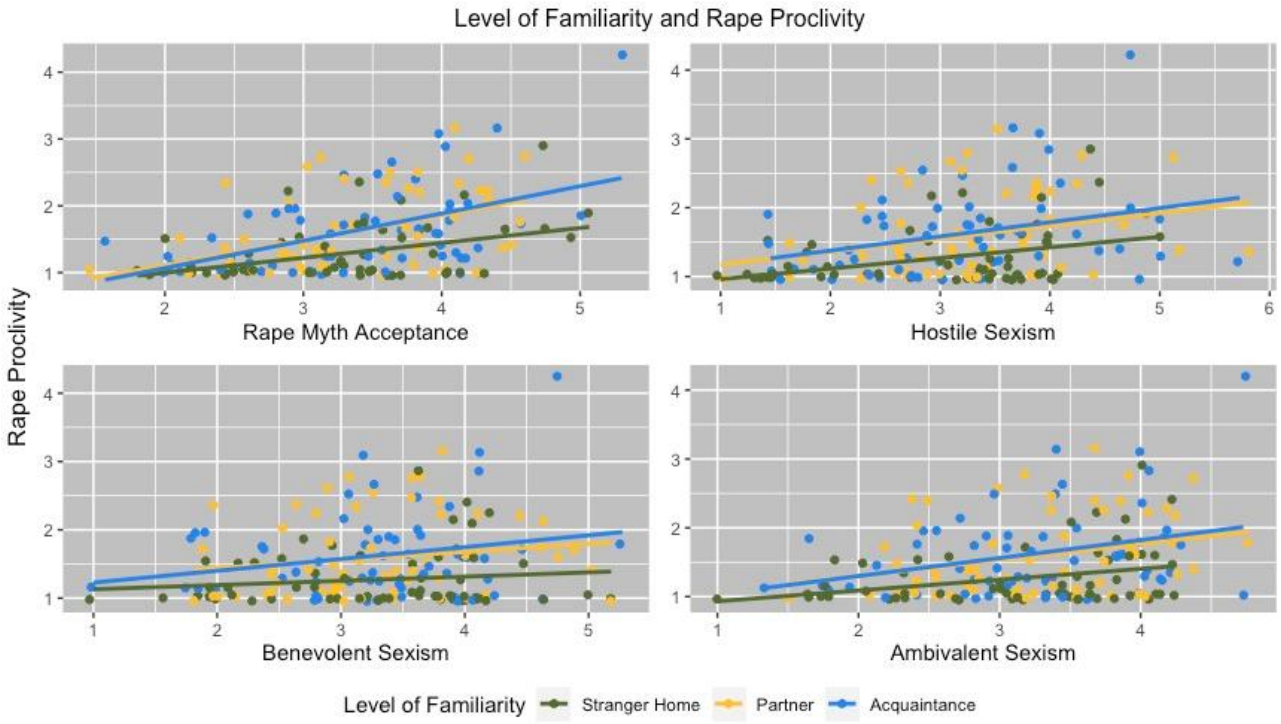

Appendix I

Tables for Study Three

Table A4

Means and Standard Deviations across Variables

	n	RP	BS	HS	RMA
Victim-perpetrator relationship					
Partner	61	1.59(0.60)	-	-	-
Acquaintance	60	1.62(0.64)	-	-	-
Stranger Home	61	1.27(0.43)	-	-	-
Total	182	1.50(0.58)	3.29(0.83)	3.15(0.96)	3.29(0.83)
